# The Auxiliary Symptoms of Early Organic Heart Disease

**Published:** 1912-09-21

**Authors:** J. Bernard Dawson


### THE AUXILIARY SYMPTOMS OF EARLY ORGANIC HEART DISEASE.

By J. BERNARD DAWSON, M.D.Lond., F.R.C.S.Eng.

Probably the most interesting thing about organic lesions of the heart is the fact that they may exist for many years without obvious symptoms. The experience of those medical men who examine for life assurance shows how often their investigation gives to the patient the first knowledge of any trouble affecting his heart, whilst there are no doubt many who die of some intercurrent disease or accident unaware of any cardiac lesion. It is a generally accepted doctrine that so long as the heart can maintain a state of compensation for its intrinsic weakness so long will symptoms of that condition remain absent. This is a dogma that has exceptions, for there are many signs of endocarditic and other lesions of the heart which may be occasionally manifest before it can be fairly said that compensation has broken down; many of these concern other organs than the heart itself.

Again, the break-down of compensation may be a very gradual process spread over months, so that there is an intermediary stage between full compensation and the invalid condition produced by the failure, which is associated with many symptoms remote from the heart itself. For the purposes of definition the term organic heart disease is intended to indicate all conditions in which there is some pathological change in either the musculature, the valves, or the coverings of the heart; so that there will be included such varied conditions as endocarditis, mitral incompetence, cardiac infarct, myocarditis of varied origin, etc.

For the purposes of classification these initial symptoms are grouped under the various systems of the body the derangement of which produces the manifestation, whilst under the vague heading of constitutional will be gathered those that cannot be definitely attributed to any system.

### The Digestive System.

Oral sepsis has sometimes a definite association with infective disorders of the heart; and although the mere existence of the unclean mouth does not, of course, indicate any cardiac condition, yet given the suspicion of endocarditis the presence of a focus of infection is in a measure a confirmation. A case which recently came under notice illustrates this. The patient, a man, had been for some weeks in bed with a persistent but intermittent temperature showing an excursion of three or four degrees. No Widal test had been tried, and the diagnosis hung between enteric fever and infective endocarditis. There were no cardiac signs other than those common to all febrile conditions. The existence of a septic sinus in the mouth pointed to the greater probability of infective valvular mischief. The agglutination test subsequently proved negative, and still later an autopsy revealed a recent and destructive lesion of the aortic and mitral valves.

Dysphagia is a rare condition which may depend upon a cardiac condition; thus in pericarditis with effusion the attention may be first attracted to the heart by the fact that there is difficulty in swallowing, and in weakly children this symptom may be the one which leads to the medical consultation. In aneurism of the thoracic aorta dysphagia may be an early symptom which, because of the alarm it causes, brings the sufferer to the doctor. Aphonia also may be produced by the interference produced by an aneurism or a pericardial effusion in the competence of the recurrent laryngeal nerve.

Dyspepsia is one of the most frequent early symptoms of heart disease. Before there is any indication of a breakdown in compensation there may be a persistent indigestion which resists all usual remedies, and an examination of the heart will reveal a valvular lesion (usually mitral incompetence). As a rule this gastric upset quickly produces a reciprocal effect upon the heart because of the flatulence and distension of the stomach which occur. It is difficult to see how this is brought about, but it may undoubtedly be manifest before there is any indication of venous stasis or pulmonary congestion. Certain cases of sudden death following a hearty meal are explained by the flatulent dyspepsia affecting a damaged heart never previously recognised. It is impossible to explain this attendant dyspepsia as a coincidence, for the trouble improves so markedly upon the exhibition of strychnine and digitalis that its association with the cardiac lesion seems definite.

### Hepatic Congestion.

This is almost invariably present after the compensation of the heart has broken down, but save for the following group of cases cannot be regarded as an auxiliary symptom of early cardiac trouble. In alcoholic subjects, however, the liver may indicate the causation of the heart weakness before any clear cardiac symptoms proper are manifest. The organic changes which appear in the hearts of alcoholic subjects usually only become manifest after there is a very definite affection of the liver and kidneys, and the large liver may be the first and most obvious finger-post which will lead to a careful investigation of the heart.

Intestinal symptoms are commonly associated with constipation and rarely with colitis. Constipation is so frequent a trouble that it cannot fairly be described as a symptom of cardiac trouble; nevertheless when a patient past middle age who has hitherto been regular in habit begins to suffer from difficulty in evacuation of the bowels it is profitable to overhaul the circulatory system. Colitis is usually an accompaniment of that stage of cardiac breakdown which produces venous stasis in the intestinal wall or as a concomitant feature of alcoholic cirrhosis of the liver, but rarely the colitis and consequent diarrhœa may appear as an early symptom of heart lesions, which are very apt to be overlooked.

The auxiliary symptoms manifested by the circulatory system concern chiefly the arterio-sclerotic group of cardiac disorders. Sclerosis of the heart and vessels produces two main and important effects—viz., thick, unyielding arteries with consequent high blood pressure and fibrotic deforming changes in the valve cusps. In this group there is a contest between the heart and the arteries as to which will stand the strain the longer; sometimes the arteries yield first, producing an apoplexy; sometimes the heart gives way, dilates, and becomes inefficient.

In the post-infective group of valvular lesions there are few early signs of mischief apparent in the circulatory system apart from the heart itself.

Persistent, intermittent, or migraine headache will sometimes direct the attention to a heart in which the valves are undergoing a sclerotic change. Syncope may occur in a case of arterial degeneration with affected aortic valve before any distress is experienced in the region of the heart; in myocarditis also syncopal attacks may be the first evidence of cardiac mischief.

A patient may be seized with angina pectoris and thus learn that degenerative changes have been taking place in the heart. An obscure case of endocarditis may be cleared up by the occurrence of emboli producing infarction of the brain, spleen, kidneys, lungs, etc.; similarly an embolus consisting of ante-mortem blood clot may occur in a pulmonary artery, with the consequent dire result.

Dyspnœa is the commonest indication of the onset of failure in compensation, and is the first that usually attracts notice both of the patient and the doctor. Shortness of breath is a valuable hint that the heart needs careful investigation, for it is an initial symptom of several degenerative changes in the myocardium; it may indicate the rise in blood pressure resulting from arterio-sclerosis, as well as suggest a breakdown in compensation. In pericarditis with effusion when the accumulation is large dyspnœa may be a consequence of the resulting pressure on the lung tissue.

A state of chronic bronchitis associated with cardiac weakness is always an interesting problem, for it is often uncertain which is the primary condition. Nevertheless there can be no doubt that a tendency to recurring attacks of bronchitis, especially during the cold season of the year, is suggestive of a failing heart. In all such cases a careful examination of the heart should be made, a precaution which will often be rewarded by the discovery that the organ is beginning to fail.

There are few associated symptoms of heart disease that are so puzzling and so interesting as those of the nervous system. Pain is often a valuable aid to diagnosis in these cases, especially when the mischief is of the sclerotic aortic variety.

Severe pain in the neck, over the manubrium sterni, in the left arm, and in the occipital region may be complained of before the patient's attention has been directed to the heart itself. In these cases investigation will often reveal the early phenomena of rising blood pressure and damaged aortic orifice.

Again in myocarditic conditions pain of every degree of severity may occur; angina pectoris being almost invariably associated with such a change.

Headache may be an early sign that all is not well with the heart, and no case of persistent headache should be dismissed without a thorough investigation, and the depressing coal-tar derivatives, so much in use as anodynes, should not be prescribed without first making sure that there is no cardiac contra-indication.

In some cases, especially those of myocarditic origin, disturbances of mental stability may occur, and although these only occur late in the disease, yet at times delusions, melancholia, and mania may occur before the heart itself has shown any symptoms obvious to the patient.

The frequent occurrence of a slight trace of albumen in a urine of low specific gravity is a very valuable aid to the investigation of an obscure case in which there are no definite signs or symptoms referable to the heart itself.

In patients past middle age this is especially valuable, for this departure from the normal kidney excretion is very common and often to be detected at an early stage of failure of cardiac efficiency. There may be some danger of confusing this urinary manifestation with that due to true chronic interstitial nephritis, which is so often followed by breakdown of the heart's power to overcome the attendant increase of peripheral resistance. A careful microscopic investigation is the only way in which this difficulty can be cleared up, and even then the presence of hyaline and granular casts in the urinary deposit from a case of a pure cardiac failure may cause confusion.

The reproductive system is one which is not often affected by cardiac changes, but there is one phenomenon which needs mention. The misadventure of repeated abortions, occurring during the early months of pregnancy, in a patient who is free from a syphilitic taint, may be an indication of a cardiac lesion. The explanation is that as soon as the added strain of pregnancy and of the growing fœtus upon the damaged maternal heart becomes too great, the stress is removed by the extrusion of the uterine contents.

Elevation of temperature may be the only indication of serious and destructive lesions of the cardiac tissue. It is a comparatively common experience for obscure cases of pyrexia to turn out to be endocarditic in origin. Anæmia is a constitutional change of some importance, partly because it is no uncommon accompaniment of early changes in the heart, and partly because anæmic conditions are so often associated with bruits in the heart that are not due to organic disease, but to a diminished density or other change in the blood.

This article is very incomplete and a mere outline of conditions which are frequently before us, which constantly present problems, and which give great difficulty in treatment. It is of vital importance that cardiac lesions should come under treatment at the earliest possible moment, and it is, therefore, important that we should have a clear conception of the fact that the first indication of heart failure is not necessarily or often a murmur or a dilatation.

